# Evaluation of Polystyrene Nanoplastics Induced Cardiotoxicity Under Different Dietary Patterns in Mice

**DOI:** 10.3390/toxics14010052

**Published:** 2025-12-31

**Authors:** Shuyi Wang, Tao Wu, Jie Dai, Xialei Liu, Lan He, Yijun Dong, Lina Zhao, Na Li

**Affiliations:** 1 School of Life Science and Technology, Wuhan Polytechnic University, Wuhan 430023, China; 2 School of Public Health, Wuhan University, Wuhan 430072, China

**Keywords:** polystyrene nanoplastics, high-fat diet, high-fructose diet, cardiotoxicity, inflammation, fibrosis

## Abstract

Background: Nanoplastics (NPs), as emerging foodborne contaminants, can accumulate in the heart and induce toxic effects. However, whether NPs exert differential cardiac impacts depending on dietary habits remains unclear. Methods: In this study, mice subjected to different dietary patterns (Normal diet, ND; High-fat diet, HFD; High-fructose diet, HFrD) were orally administered 80 nm polystyrene nanoplastics (PS-NPs) at a dose of 10 mg/(kg·day) for 1, 4, and 8 weeks. The fluorescence tracing, histopathological analysis, quantification of inflammatory and fibrotic markers, and transcriptomic sequencing were used to evaluate the distribution and hazardous effect of PS-NPs. Results: By the 8th week, significant fluorescence labeled PS-NPs accumulation was detected in the hearts of mice on HFD group and HFrD group. Histopathological and immunofluorescence analyses revealed that both HFD and HFrD groups exacerbated cardiac collagen deposition and inflammatory infiltration in PS-NP-exposed mice. Transcriptomic analysis further indicated that under HFD, PS-NP exposure primarily activated MAPK signaling pathway-mediated inflammation, thereby promoting fibrosis. In contrast, under HFrD, PS-NP80 amplified cardiac injury via the TNF signaling pathway. Conclusions: These findings demonstrate that dietary habits can aggravate the cardiac toxicity induced by foodborne nanoplastics, highlighting the importance of considering dietary patterns in the risk assessment of food contaminants.

## 1. Introduction

Over recent decades, plastic products have been extensively utilized in food packaging, kitchenware, and other fields. Under natural conditions such as photo-oxidation, mechanical abrasion, and biodegradation, plastic items break down into microplastics (MPs, diameter < 5 mm), and further into nanoplastics (NPs, diameter < 1 μm) [1,2]. The estimated daily intake of plastic particles in humans ranges from 0.23 to 11.9 mg/kg, with oral exposure being the primary route [3,4]. MPs released from plastic containers enter the human body through beverages and food, posing a potential health risk as emerging foodborne contaminants. Among them, polystyrene nanoplastics (PS-NPs)—a common polymer widely used in food packaging such as takeaway boxes, food containers, and disposable cups and cutlery [5]—can accumulate in the body when not efficiently excreted. Recent studies have detected PS-NPs in multiple organs, including the gastrointestinal tract, liver, and heart [6,7,8], highlighting the need for further investigation into the health risks of dietary PS-NPs.

The heart, as a vital circulatory organ, is highly sensitive to various injurious factors. In recent years, the link between food contaminants and cardiotoxicity has drawn increasing attention. Experimental studies suggest that chronic exposure to heavy metals and chemical toxins can induce cardiotoxicity by activating inflammatory signaling and oxidative stress [9]. As an emerging foodborne risk factor, PS-NPs have been shown to accumulate in cardiac tissue, leading to mitochondrial dysfunction, excessive release of reactive oxygen species (ROS), and enrichment of inflammatory factors [10,11,12,13]. These processes impair myocardial contraction and relaxation, disrupt normal cardiac structure and function, and ultimately trigger cardiotoxic responses.

Dietary patterns significantly influence physiological states and disease susceptibility by modulating processes such as inflammation, oxidative stress, and tissue repair [14,15,16]. Previous studies have confirmed that different diets can alter the body’s sensitivity to environmental pollutants. For example, dietary restriction was shown to exacerbate PS-MPs-induced toxicity, leading to intestinal barrier dysfunction and liver injury [17]. MPs combined with a high-fat diet have been reported to worsen gut leakiness and inflammation [18], and NPs have been linked to glomerular damage and renal tubulointerstitial fibrosis in mice on a high-fat diet [19]. However, it remains unclear how different dietary patterns influence the cardiac accumulation and toxicity of nanoplastics, and the underlying mechanisms have not yet been elucidated.

Therefore, this study aims to investigate the differences in cardiac accumulation and toxicity of PS-NPs under different dietary regimens—normal diet (ND), high-fat diet (HFD), and high-fructose diet (HFrD). We established mouse models exposed to PS-NPs for 1, 4, and 8 weeks under each dietary condition. Using fluorescence tracing, histopathological analysis of cardiac tissue, quantification of inflammatory and fibrotic markers, and transcriptomic sequencing, we comprehensively evaluated the differential cardiac accumulation, toxicity, and potential mechanisms of PS-NPs across dietary patterns. Our findings may provide experimental evidence for cardiotoxicity assessment of nanoplastics and the development of dietary intervention strategies.

## 2. Materials and Methods

### 2.1. PS-NPs Characterization

Fluorescently labeled polystyrene nanoplastics (PS-NPs) and original PS-NPs were acquired from Tianjin BaseLine ChromTech Research Centre (Tianjin, China). Fluorescent labeled PS-NPs were used to label the accumulation of NPs in the heart, while the original PS-NPs were used for in vivo toxicity testing. After diluting the PS-NPs stock solution with distilled water, the morphology of PS-NPs was evaluated using a scanning electron microscope (SEM) (Zeiss Gemini SEM 500, Oberkochen, Germany) [17], and the hydration particle size and Zeta potential of PS-NPs were measured using a Malvern nanoparticle potential analyzer.

### 2.2. Experimental Animal Grouping and Treatment

The C57BL/6J mice (three weeks, male) were obtained from Shulaibao Biotechnology Co., Ltd. (Chongqing, China) and kept in a specific pathogen-free environment under controlled temperature at 22 ± 2 °C, humidity at 50–60%, and 12 h light/dark cycles. Each mouse was freely accessible to diets and water. All animal experimental procedures were approved by the Animal Ethics Committee of Wuhan University (Approval No. WP20240363). After 1 week of acclimation, mice were continuously administered fluorescent and non-fluorescent polystyrene nanoplastics (PS-NPs) were vibrated by ultrasound for 20 min and then diluted with deionized water for mice via oral gavage for 1, 4, and 8 weeks at a dose of 10 mg/(kg·bw·day) [20] fed with normal, high-fat (60% High-Fat Diet, D12492) or high-fructose diet (30% Fructose Water), control group received normal chow, normal drinking water, and an equal volume of deionized water administered via daily oral gavage. It has been previously reported that the daily intake of plastic particles in humans weighing 60 kg is about 0.04–11.7 mg/kg [21]. Dose conversion between humans and mice was performed based on body surface area using the Km factor method [22]. In our experiment, we chose a dosage of NPs of 0.5 mg/d, which is 10 mg/(kg·body weight·day).

### 2.3. Ex Vivo Fluorescence Imaging

Following 1-, 4-, and 8-week exposure to fluorescent polystyrene nanoplastics (PS-NPs), mice were fasted for 12 h, anesthetized with isoflurane (1.5–2.0%), and euthanized. Hearts were excised, and to minimize the autofluorescence of red blood cells to the greatest extent, the mouse hearts were perfused with PBS to eliminate residual blood prior to tissue imaging. Subsequently, the perfused hearts were placed on black paper and subjected to ex vivo fluorescence imaging for signal acquisition. Fluorescence images were captured using a 450 nm excitation filter and a 680 nm emission filter (PerkinElmer, IVIS SPECTRUM, Shelton, CT, USA). Fluorescence intensity was quantified using Living Image software (version 4.4).

### 2.4. Histopathological Examination in Heart Tissue

Following the completion of oral exposure, mice were anesthetized with isoflurane and euthanized. Cardiac tissues were promptly collected and fixed overnight in 4% paraformaldehyde. The fixed tissues were then dehydrated through a graded ethanol series, embedded in paraffin, and sectioned into 4 μm thick slices. After deparaffinization, the sections were stained with hematoxylin and eosin (H&E) (Servicebio, Wuhan, China), dehydrated, mounted, and finally observed under an optical microscope [23] (Nikon, E100, Shinagawa, Japan).

### 2.5. Masson’s Trichrome and Sirius Red Staining

Paraffin-embedded tissue blocks were sectioned into 4 μm thick slices, deparaffinized and rehydrated. The sections were subsequently stained with Masson’s Trichrome stain (Servicebio, Wuhan, China) and Sirius Red stain (Servicebio, Wuhan, China), respectively. After staining, the sections were cleared and mounted with neutral resin. Cardiac tissue sections were then examined under a microscope (Nikon, E100, Shinagawa, Japan) to observe histological changes and assess the degree of fibrosis. For each section, five random fields were selected, and the integrated optical density (IOD) of each field was quantified using Image J software v1.54r [23].

### 2.6. Enzyme Linked Immunosorbent Assay (ELISA) Detection of CK-MB, Cardiac Troponin T, TNF-α and IL-1β

Quantify the levels of creatine kinase-MB (CK-MB) and cardiac troponin T (cTnT) in serum using an ELISA kit [24,25] (Jingmei, Jiangsu, China; CK-MB Catalog #JM-03084M1; Cardiac troponin T Catalog #JM-11710M1). Additionally, determine the levels of tumor necrosis factor-*α* (TNF-*α*) and interleukin-1*β* (IL-1*β*) in cardiac tissue of mice at the 8th week using corresponding ELISA kit (Jingmei, Jiangsu, China; TNF-*α* Catalog #JM-02415M1; IL-1*β* Catalog #JM-02323M1). The experimental plan is based on the manufacturer’s instructions. After adding the standard and sample to each well, the prepared biotin antibody was added and incubated at 37 °C for 30 min. The substrate reagent was added and incubated for 10 min [26]. The termination solution was quickly added and read using a microplate reader (Tecan Infinite 200 PRO, CH, Zurich, Switzerland).

### 2.7. Immunohistochemical Staining of Collagen I and Collagen III

After deparaffinization, paraffin sections underwent antigen retrieval followed by three washes with PBS. The sections were blocked with 3% BSA at room temperature for 30 min, then incubated overnight at 4 °C with diluted primary antibodies (Details of the primary antibody used in this experiment are provided in Table 1) against Collagen I and Collagen III. Subsequently, the sections were incubated with goat anti-rabbit IgG secondary antibody (Servicebio, HRP- labeled, dilution ratio 1:500) at room temperature for 50 min. After slight drying, freshly prepared DAB substrate was applied to the marked areas. Nuclei were counterstained with hematoxylin (Servicebio, Wuhan, China) for approximately 3 min, followed by dehydration, clearing, and mounting. Sections were observed and imaged under an optical microscope (Nikon, E100, Shinagawa, Japan), with five random fields captured per section. The integrated optical density (IOD) was quantified using Image J software to analyze Collagen I and Collagen III positive expression.

### 2.8. Immunofluorescence Staining for TNF-α and IL-1β

After deparaffinization and rehydration, paraffin sections were subjected to antigen retrieval. The sections were then washed three times with PBS and blocked with 3% BSA at room temperature for 30 min. Subsequently, the sections were incubated overnight at 4 °C with diluted primary antibodies (Details of the primary antibody used in this experiment are provided in Table 1) against TNF-α and IL-1β. This was followed by incubation with goat anti-rabbit IgG secondary antibody (Servicebio, CY3- labeling, Excitation wavelength: 510–560 nm, Emission wavelength: 590 nm, dilution ratio 1:300) at room temperature for 50 min. The sections were then stained with DAPI solution for 10 min at room temperature in the dark. Finally, the sections were mounted with anti-fade mounting medium. Imaging was performed using a fluorescence microscope (Nikon, Nikon Eclipse C1, Shinagawa, Japan), with five random fields captured per section. Fluorescence intensity was quantified using Image J software.

### 2.9. RNA Sequencing Analysis

Three heart samples from each group after 8 weeks of exposure were selected for RNA sequencing. Total RNA was extracted from the tissue using Trizol (Invitrogen, Carlsbad, CA, USA) according to the manufacturer’s instructions. The quality and quantity of total RNA were determined using an Agilent 2100 Bioanalyzer (Agilent, Santa Clara, CA, USA). Then, the library was prepared using the BGI Optimal series dual-module mRNA library construction kit (BGI-Shenzhen, Shenzhen, China) and sequenced on the T7 sequencer (BGI-Shenzhen, Shenzhen, China) with PE150 sequencing. The raw reads in fastq format were first processed using Trimmomatic, and the clean data were aligned to the mouse genome (Mus_musculus.GRCm39) using HISAT2-2.1.0 software [27]. Differentially expressed genes (DEGs) were identified using DESeq2 (v1.34.0) [28] with the criteria of Q value ≤ 0.05 or FDR ≤ 0.001. To further explore gene functions related to phenotypic changes, KEGG enrichment analysis (https://www.kegg.jp/, accessed on 25 September 2025) was performed on the DEGs using Phyper based on the hypergeometric test, with a threshold of Q value ≤ 0.05. Terms meeting this condition were defined as significantly enriched in the candidate genes.

### 2.10. Western Blotting Analysis

Heart tissues were ground and lysed in RIPA lysis buffer (Beyotime, Shanghai, China) supplemented with PMSF protein inhibitor (Servicebio, China) and phosphatase inhibitor (Servicebio, China). The supernatant was collected after centrifugation at 4 °C, and its protein concentration was determined using a BCA protein assay kit (Beyotime, China). Following separation via 10% SDS-PAGE gel electrophoresis, the proteins were transferred onto a PVDF membrane, which was then blocked with 5% skimmed milk at room temperature for 2 h. The membrane was subsequently incubated with primary antibodies (Details of the primary antibody used in this experiment are provided in Table 1) at 4 °C for 16 h. After washing with TBST, the membrane was incubated with secondary antibody dilution (HUABIO, HA1001, dilution ratio 1:100,000, Hangzhou, China) at room temperature for 1.5 h. Image acquisition was performed using the imaging system (Bio-Rad, Gel Doc XR+ Gel, Hercules, CA, USA), and grayscale analysis of the results was conducted using Image J software.

### 2.11. Statistical Analysis

Statistical analysis was conducted with GraphPad Prism 9. Differences among groups were evaluated through one-way ANOVA or two-way ANOVA, with suitable post hoc tests applied. The results are shown as the means ± SEMs. An alpha threshold of 0.05 was used, and *p* values below this threshold were regarded as significant.

## 3. Results

### 3.1. Characterization of PS-NPs

**Figure 1:**
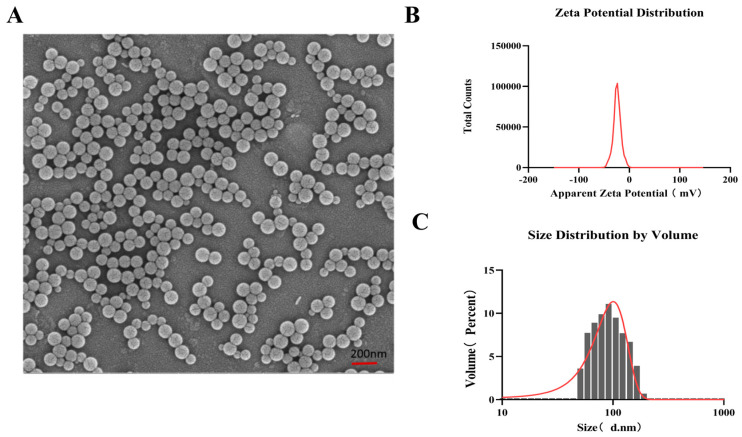
Characterization of PS MNPs. (**A**) Representative SEM images of PS-80 (left, scale bar = 200 nm). (**B**) Zeta potential of PS-80 in ddH_2_O. (**C**) Hydrodynamic size distribution of PS-80 in ddH_2_O.

To verify the characteristics of the PS-NPs used in this study, the PS-NPs employed in the experiment were characterized. Scanning electron microscopy revealed that the PS-NPs used in this experiment exhibited regular, uniform spherical particles (Figure 1A). The Zeta potential of PS-NPs dissolved in pure water was −25.55 mV, indicating that the PS-NPs used in this study exhibit good stability and dispersibility (Figure 1B). The hydrated particle size of the PS-NPs used in this experiment was measured to be 80.8 nm (Figure 1C).

### 3.2. Distribution of PS-NPs in Mouse Heart Tissue In Vivo

**Figure 2:**
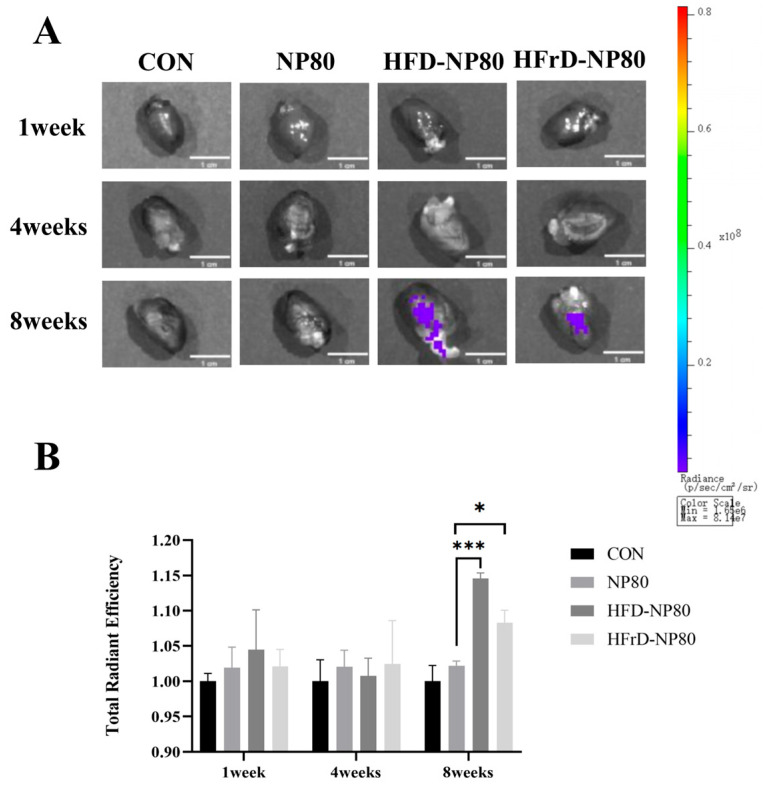
Distribution of fluorescent PS-NPs in the heart. (**A**) Fluorescence PS-NPs distribution in the heart after 1, 4, and 8 weeks of exposure in different groups (*n* = 3/group). (**B**) Quantitative statistical results of fluorescence intensity at 1, 4, and 8 weeks in different groups. The data are expressed as mean ± SEM (* *p* < 0.05, *** *p* < 0.001).

The heart is one of the most important organs in the human body, whose primary function is to generate the force required for blood circulation [11]. To investigate whether different dietary patterns influence the accumulation of PS-NPs under the same exposure conditions, we administered red fluorescently labeled PS-NPs via gavage and observed their accumulation in mouse hearts at different time points. As shown in Figure 2A, no significant accumulation of fluorescent PS-NPs was observed in the hearts of mice in the normal diet combined with PS-NPs exposure group at 1, 4, and 8 weeks post-exposure. However, significant fluorescent PS-NPs accumulation was observed in the hearts of mice in both the high-fat diet combined with PS-NPs exposure group (HFD-NP80) and the high-fructose diet combined with PS-NPs exposure group (HFrD-NP80) at 8 weeks (Figure 2B). These results indicate that under prolonged PS-NPs exposure, high-fat and high-fructose dietary patterns promote the accumulation of PS-NPs in the heart.

### 3.3. General Physical Conditions of Mice

**Figure 3:**
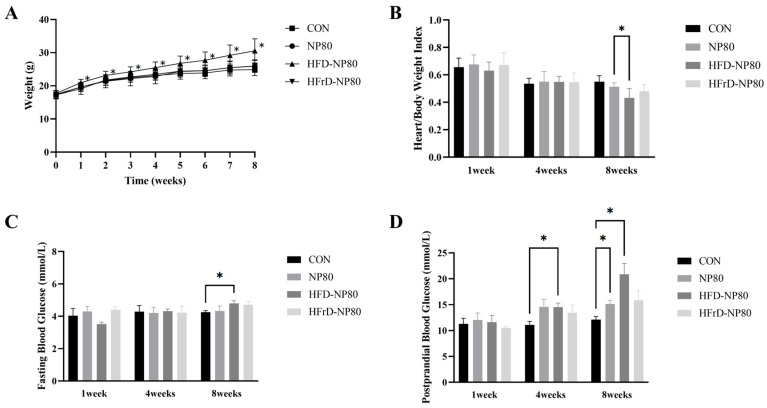
General Physical Conditions of Mice. (**A**) Body weight of mice throughout the experiment. (**B**) Fasting blood glucose levels in mice. (**C**) Postprandial blood glucose levels in mice. (**D**) Organ coefficient levels of the liver. The data are expressed as mean ± SEM (* *p* < 0.05).

As described in Figure 3A, the body weight of mice in the high-fat diet group was significantly higher than that in the other groups throughout the experimental period. At the 8th week, compared with the NP80 group, the ratio of heart weight to body weight in the HFD-NP80 group decreased significantly (Figure 3B). As shown in Figure 3C, the fasting blood glucose level of mice in the high-fat diet group was significantly higher than that in the CON group at the 8th week. In contrast, differences in postprandial blood glucose levels emerged from the 4th week; as shown in Figure 3D, the postprandial blood glucose level of mice in the high-fat diet group was significantly higher than that in the CON group at the 4th week. At the 8th week, the postprandial blood glucose levels of mice in the PS-NPs exposure group and the high-fat diet combined with PS-NPs exposure group were significantly higher than those in the CON group.

### 3.4. Myocardial Pathological Changes In Mice Treated with PS-NPs Under Different Dietary Patterns

**Figure 4:**
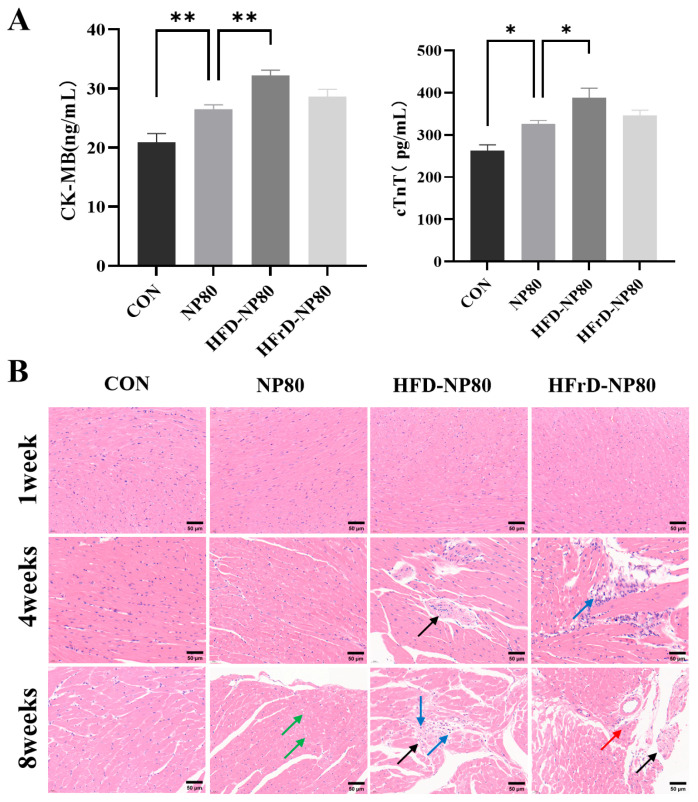
Effects of PS-NPs on cardiac injury under different dietary patterns. (**A**) The level of CK-MB (Left) and the level of Cardiac Troponin T (Right) (*n* = 6/group). The data are expressed as mean ± SEM (* *p* < 0.05, ** *p* < 0.01). (**B**) H&E staining of mouse cardiac tissue (*n* = 4/group).

CK-MB and cTnT are two specific markers of myocardial injury in clinical settings [29]. This study demonstrates that PS-NPs 80 nm significantly elevate the levels of CK-MB and cTnT in myocardial cells. In the HFD-NP80 group, CK-MB and cTnT levels were significantly higher than those in the NP80 group (Figure 4A). Although there was no significant difference between the HFrD-NP80 group and the NP80 group, CK-MB and cTnT levels were still elevated by 1.08-fold and 1.06-fold, respectively.

Myocardial pathological changes in mice by H&E staining revealed that in the control group, the heart tissue structure of mice was clear, with distinct cell boundaries and no abnormalities in the interstitium (Figure 4B). In contrast, mice in the NP80 group exhibited mild vacuolization of myocardial cells (green arrows) after 8 weeks of intervention. In the HFD-NP80 group, interstitial vascular damage was observed in the myocardial tissue at the 4th week, characterized by thickened vessel walls, narrowed lumens, irregularly arranged smooth muscle cells with indistinct boundaries (black arrows). At the 8th week, mild hyperplasia of connective tissue was seen in multiple locations (black arrows), accompanied by a small amount of lymphocyte infiltration (blue arrows). In the HFrD-NP80 group, small areas of irregularly arranged myocardial cells and mild hyperplasia of interstitial connective tissue were observed in the mice’s hearts at the 4th week, with occasional lymphocyte infiltration (blue arrows). By the 8th week, mild myocardial cell necrosis (red arrows) was observed, characterized by pyknosis and deep staining, dissolution and disappearance of nuclei, increased eosinophilia of the cytoplasm, and occasional mild hyperplasia of connective tissue (black arrows).

These findings indicate that NPs under different dietary patterns induced varying degrees of myocardial damage in the myocardial tissue. Moreover, with prolonged intervention time, high-fat and high-fructose diets exacerbated inflammatory cell infiltration, myocardial cell vacuolization, and connective tissue hyperplasia under PS-NP80 intervention.

### 3.5. Fibrosis Analysis of Cardiac Tissue

**Figure 5:**
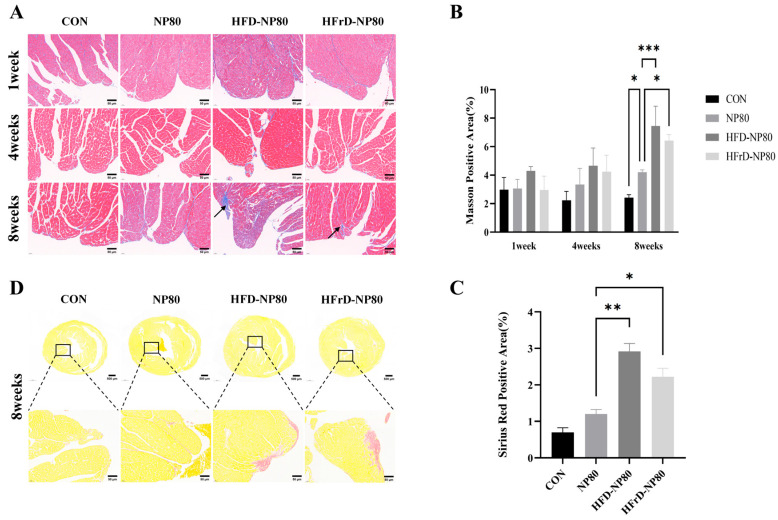
Effects of PS-NPs on cardiac tissue structure and tissue collagen expression under different dietary patterns. (**A**) Masson’s trichrome staining of mouse cardiac tissue (*n* = 4/group). (**B**) Quantification of collagen positive Masson staining. (**C**) Sirius red staining of mouse cardiac tissue (*n* = 4/group). (**D**) Quantification of collagen positive Sirius red staining. The data are expressed as mean ± SEM (* *p* < 0.05, ** *p* < 0.01, *** *p* < 0.001).

Additionally, Masson staining showed that, after 8 weeks of continuous intervention, NP80 group exhibited increased collagen deposition relative to the CON group. Moreover, a significant increase in collagen deposition was observed in the HFD-NP80 and HFrD-NP80 groups compared to the NP80 group (Figure 5A,B), suggesting that prolonged high-fat and high-fructose diets exacerbate fibrosis in heart tissue under PS-NPs intervention. To verify this viewpoint, Sirius red staining was performed on the heart tissues of mice at the 8th week, and the results demonstrated that after PS-NP80 treatment, HFD-NP80 and HFrD-NP80 groups could induce collagen deposition in the mouse hearts (Figure 5C,D).

### 3.6. Collagen and TGF-β1/Smad2 Pathway Expression of PS-NPs Exposure on Cardiac Fibrosis

**Figure 6:**
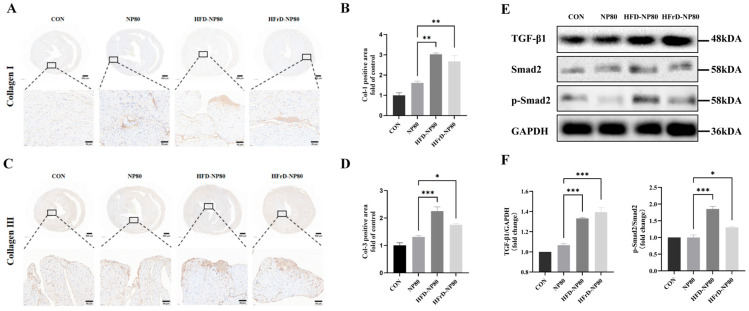
High-fat/high-fructose diet exacerbates PS-NP-mediated collagen deposition leading to cardiac fibrosis. (**A**) Representative IHC images and (**B**) quantitative analysis of Collagen I expression in mouse heart tissues at the 8th week (*n* = 4/group). (**C**) Representative IHC images and quantitative analysis (**D**) of Collagen III expression in mouse heart tissues at the 8th week (*n* = 4/group). (**E**) The protein expressions and quantitative analysis (**F**) of TGF-β, Smad2 and p-Smad2 in mouse heart tissues at the 8th week by Western blotting assay. GAPDH is used as internal references (*n* = 4/group). The data are expressed as mean ± SEM (* *p* < 0.05, ** *p* < 0.01, *** *p* < 0.001).

To characterize the type of collagen deposition, we performed immunohistochemical staining for Collagen I and Collagen III on cardiac tissues collected at week 8. The results showed that the expression of both Collagen I (Figure 6A,B) and Collagen III (Figure 6C,D) was significantly increased in the myocardial tissues of HFD-NP80 and HFrD-NP80 mice compared with the NP80 group. These findings indicate that high-fat or high-fructose diets lead to the deposition of Collagen I and Collagen III in the myocardium, thereby promoting the progression of cardiac fibrosis.

The TGF-β1/Smad2 signaling pathway is considered to be the main factor regulating collagen synthesis and deposition. We detected the expression of the TGF-β1/Smad2 signaling pathway in the cardiac tissues of mice at the 8th week. Compared with the NP80 group, the expression levels of TGF-β1 and phosphorylated Smad2 were significantly up-regulated in the HFD-NP80 and HFrD-NP80 groups (Figure 6E,F), indicating that the TGF-β1/Smad2 signaling pathway was highly activated in the myocardial tissues of mice exposed to PS-NPs for eight weeks under high-fat diet and high-fructose diet conditions. These findings indicate that high-fat or high-fructose diets lead to the synthesis and deposition of Collagen I and Collagen III in the myocardium by activating the TGF-β1/Smad2 signaling pathway, thereby promoting the progression of cardiac fibrosis.

### 3.7. Effects of PS-NPs on the Inflammatory Response Level in Mouse Cardiac Tissue

**Figure 7:**
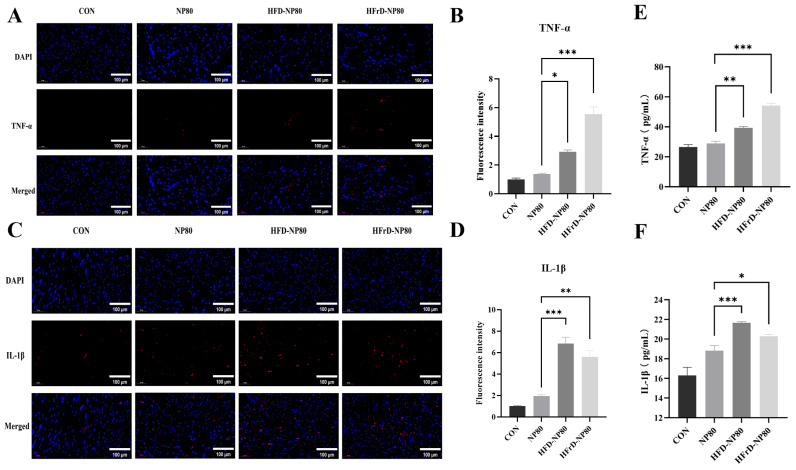
Effects of PS-NPs on the inflammatory response level in mouse cardiac tissue under diverse dietary patterns. (**A**) IF analysis of TNF-α expression and quantification of fluorescence intensity (**B**) in the heart tissue of mice at the 8th week (*n* = 4/group). (**C**) IF analysis of IL-β expression and quantification of fluorescence intensity (**D**) in the heart tissue of mice at the 8th week (*n* = 4/group). (**E**,**F**) Enzyme-linked immunosorbent assay (ELISA) analysis of the concentrations of TNF-α and IL-1β in the heart tissue of mice at the 8th week (*n* = 4/group). The data are expressed as mean ± SEM (* *p* < 0.05, ** *p* < 0.01, *** *p* < 0.001).

Inflammation is one of the key factors leading to cardiac fibrosis. H&E staining further revealed that under different dietary patterns, PS-NPs induced varying degrees of inflammatory cell infiltration. Immunofluorescence analysis revealed that at the eighth week, the expression levels of TNF-α (Figure 7A,B) and IL-1β (Figure 7C,D) in myocardial tissue of mice in the HFD-NP80 group were significantly elevated compared to those in the NP80 group. Meanwhile, significant differences were observed in the expression of IL-1β in cardiac tissues between the HFrD-NP80 and NP80 groups. Further evaluation of TNF-α and IL-1β expression levels in cardiac tissue via ELISA revealed similar trends (Figure 7E,F). These findings indicate that under different dietary regimens, nanoparticles (NPs) induce varying degrees of inflammatory injury in myocardial tissue, with a more severe inflammatory response triggered by NPs under a high-fat diet.

### 3.8. Transcriptome Analysis of PS-NP-Exposed Mouse Hearts Under Different Dietary Patterns

**Figure 8:**
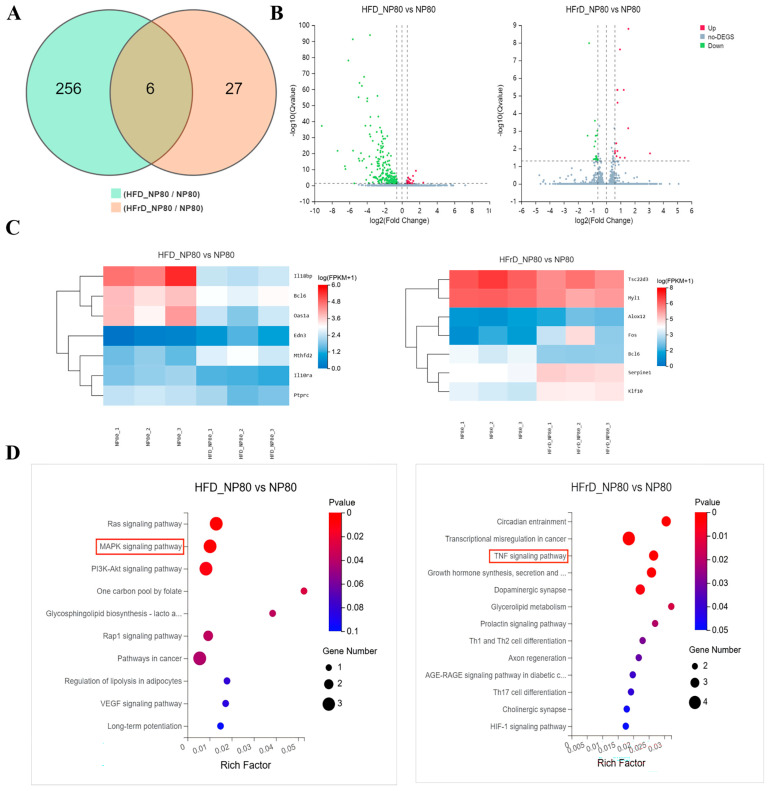
Transcriptome analysis of mouse heart tissue following 8 weeks of PS-NPs exposure under different dietary patterns. (**A**) Venn diagram of DEGs following PS-NPs exposure under different dietary patterns (*n* = 3/group). (**B**) Volcano plot of DEGs following PS-NPs exposure under different dietary patterns. (**C**) Heatmap of selected genes with differential expression in both experimental and control groups. (**D**) KEGG pathways were enriched based on the DEGs from the NP80 group.

To elucidate the potential mechanisms underlying the differences in cardiotoxicity induced by PS-NPs exposure under different dietary patterns, we performed RNA sequencing on mouse heart tissues at the eighth week. As shown in the Venn diagram, compared with the NP80 group, 262 differentially expressed genes were identified in the HFD-NP80 group, while 33 differentially expressed genes were identified in the HFrD-NP80 group (Figure 8A). Volcano plots revealed that the high-fat diet led to the up-regulation of 21 genes and down-regulation of 241 genes. The high-sugar diet resulted in the up-regulation of 15 genes and down-regulation of 18 genes (Figure 8B). Clustering heatmaps further illustrated the mRNA expression levels. Compared with the NP80 group, the pro-fibrotic gene *Edn3* and pro-inflammatory gene *Mthfd2* were significantly up-regulated in the HFD-NP80 group, while anti-inflammatory genes *Il18bp*, *Il10ra*, and *Bcl6* were significantly down-regulated. In the HFrD-NP80 group, genes promoting inflammation and fibrosis, such as *Alox12*, *Fos*, and *Serpine1*, were significantly up-regulated, whereas anti-inflammatory genes including *Bcl6* and *Tsc22d3* were significantly down-regulated (Figure 8C).

KEGG enrichment analysis revealed distinct pathways enriched after 8 weeks of PS-NPs intervention under different dietary patterns. The MAPK signaling pathway was significantly enriched in the HFD-NP80 group (Figure 8D), while the TNF signaling pathway was significantly enriched in the HFrD-NP80 group. The TNF signaling pathway rapidly initiates and amplifies inflammatory genes through the TNFR1–NF-κB/MAPK axis. These results suggest that PS-NPs under different dietary patterns may activate distinct signaling pathways, leading to varying degrees of inflammatory responses and their amplification, which in turn induces myocardial fibrosis and ultimately differences in cardiotoxicity.

## 4. Discussion

As the application of plastic products becomes increasingly widespread in daily life, the pollution caused by micro- and nanoplastics (MNPs) and its impact on human health have increasingly become a focus of attention among researchers. Current research on the interactive effects of MNPs and different dietary patterns on tissues and organs has primarily focused on the intestine [30,31], liver [31], adipose tissue [32], reproductive organs [33], and kidneys [19]. The heart, being a high-energy-consuming and high-metabolic organ, is not only directly exposed to circulating MNPs [34] but is also profoundly influenced by dietary patterns, making it a potential “common target” for the synergistic effects of MNPs and different diets. However, there remains a paucity of research on the cardiac toxicity of MNPs under various dietary conditions and its associated molecular mechanisms.

In this study, male C57BL/6 mice were used and divided into control, normal diet-NP80, high-fat diet-NP80, and high-fructose diet-NP80 exposure groups. The toxicological differences in PS-NPs on the heart under different dietary patterns were evaluated using a combination of histopathological, biochemical, and transcriptomic analyses. Initially, we observed significant accumulation of PS-NPs in the heart tissues of mice on high-fat and high-fructose diets by week 8, possibly due to impaired intestinal barrier function induced by a high-fat diet (HFD) [18,35] or intestinal metabolic disorders caused by a high-fructose diet (HFrD) [36], which increased the absorption of PS-NPs and/or altered their distribution kinetics in vivo. In contrast, no significant accumulation of PS-NPs was found in the hearts of mice on a normal diet, suggesting that a normal diet helps maintain intestinal barrier integrity, thereby limiting the entry of PS-NPs into the bloodstream.

Elevated serum levels of CK-MB and cTnT are specific markers of myocardial cell injury. In this study, both high-fat and high-fructose dietary patterns exacerbated PS-NPs-induced myocardial injury, consistent with the observed myocardial cell degeneration, necrosis, and inflammatory infiltration in heart HE staining. Cardiac fibrosis, a consequence of long-term tissue damage, has been previously reported to occur with significant collagen deposition after oral exposure to 30 mg/L PS-NPs for 6 weeks in mice [37]. By week 8 of exposure in our study, high-fat and high-fructose diets variously aggravated the deposition of type I/III collagen in heart tissue under PS-NPs exposure by activating the TGF-β1/Smad2 signaling pathway. Inflammation is a core inducer of cardiac fibrosis [38], and immunofluorescence staining for inflammatory cytokines TNF-α and IL-1β revealed that high-fat and high-fructose diets promoted the secretion of these cytokines due to PS-NPs exposure.

To explore the specific molecular mechanisms underlying the differential cardiac toxicity of PS-NPs under different dietary patterns, our transcriptomic analysis revealed that high-fat and high-fructose diets amplified inflammatory responses by activating distinct signaling pathways. The differentially expressed genes in the HFD-NP80 group were enriched in the MAPK signaling pathway, a crucial component of the immune response. Activation of the MAPK pathway triggers the release of numerous inflammatory cytokines from macrophages. Previous studies have shown that elevated levels of free fatty acids (FFAs) under high-fat diets can activate the MAPK signaling pathway in the heart, initiating an inflammatory response that leads to myocardial hypertrophy and fibrosis [39]. Conversely, in the HFrD-NP80 group, differentially expressed genes were enriched in the TNF signaling pathway, with TNF-α, a key cytokine in this pathway, promoting the expression of inflammatory cytokines by activating the downstream NF-κB signaling pathway through binding to TNFR1 [40]. Cardiac fibrosis, a late outcome of prolonged PS-NPs exposure, is closely associated with the intensity of the inflammatory response. Differential regulation of inflammatory cytokines by different dietary patterns can enhance the cardiac toxicity induced by PS-NPs exposure, activating the transformation of cardiac fibroblasts into myofibroblasts, further stimulating the TGF-β pathway [41], promoting the expression of type I and III collagen, and advancing the fibrotic process, forming a positive feedback loop of “inflammation-fibrosis.”

Nevertheless, our study has limitations. Firstly, the absence of separate high-fat and high-fructose diet control groups makes it impossible to rule out whether the toxic effects of the diets themselves on the heart exceed those of PS-NPs. Secondly, the lack of detection of protein expression and phosphorylation levels of key molecules in the signaling pathways hinders the clarification of the cascade reactions of pathway activation. Finally, the fibrotic pathways were not further explored.

## 5. Conclusions

In summary, our study demonstrates that high-fat and high-fructose diets can promote the accumulation of PS-NPs in the heart and exacerbate their cardiotoxic effects. However, the mechanisms underlying the differential toxicity induced by different dietary patterns differ substantially.

## Figures and Tables

**Table 1:** Primer antibodies used.

Antibody Name	Dilution Ratio	Resource
Collagen I	1:1000	Servicebio
Collagen III	1:500	Servicebio
TNF-α	1:1000	Servicebio
IL-1β	1:1000	Servicebio
TGF-β1	1:2000	HUABIO
Smad2	1:1000	HUABIO
p-Smad2	1:1000	HUABIO

## Data Availability

The datasets supporting the conclusions of this article are included within the article and can be retrieved from the corresponding author upon reasonable request.
